# Further Evidence for Bats as the Evolutionary Source of Middle East Respiratory Syndrome Coronavirus

**DOI:** 10.1128/mBio.00373-17

**Published:** 2017-04-04

**Authors:** S. J. Anthony, K. Gilardi, V. D. Menachery, T. Goldstein, B. Ssebide, R. Mbabazi, I. Navarrete-Macias, E. Liang, H. Wells, A. Hicks, A. Petrosov, D. K. Byarugaba, K. Debbink, K. H. Dinnon, T. Scobey, S. H. Randell, B. L. Yount, M. Cranfield, C. K. Johnson, R. S. Baric, W. I. Lipkin, J. A. K. Mazet

**Affiliations:** a Center for Infection and Immunity, Mailman School of Public Health, Columbia University, New York, New York, USA; b Department of Epidemiology, Mailman School of Public Health, Columbia University, New York, New York, USA; c EcoHealth Alliance, New York, New York, USA; d One Health Institute and Karen C. Drayer Wildlife Health Center, School of Veterinary Medicine, University of California, Davis, California, USA; e Department of Microbiology and Immunology, University of North Carolina School of Medicine, Chapel Hill, North Carolina, USA; f Gorilla Doctors, c/o MGVP, Inc., Davis, California, USA; g Makerere University Walter Reed Project, Kampala, Uganda; h Makerere University, College of Veterinary Medicine, Kampala, Uganda; i Department of Epidemiology, University of North Carolina School of Medicine, Chapel Hill, North Carolina, USA; j Department of Cell Biology and Physiology and Marsico Lung Institute/Cystic Fibrosis Center, University of North Carolina School of Medicine, Chapel Hill, North Carolina, USA; St. Jude Children's Research Hospital

**Keywords:** bat, MERS coronavirus, spike, Uganda, zoonoses

## Abstract

The evolutionary origins of Middle East respiratory syndrome (MERS) coronavirus (MERS-CoV) are unknown. Current evidence suggests that insectivorous bats are likely to be the original source, as several 2c CoVs have been described from various species in the family *Vespertilionidae*. Here, we describe a MERS-like CoV identified from a *Pipistrellus cf. hesperidus* bat sampled in Uganda (strain PREDICT/PDF-2180), further supporting the hypothesis that bats are the evolutionary source of MERS-CoV. Phylogenetic analysis showed that PREDICT/PDF-2180 is closely related to MERS-CoV across much of its genome, consistent with a common ancestry; however, the spike protein was highly divergent (46% amino acid identity), suggesting that the two viruses may have different receptor binding properties. Indeed, several amino acid substitutions were identified in key binding residues that were predicted to block PREDICT/PDF-2180 from attaching to the MERS-CoV DPP4 receptor. To experimentally test this hypothesis, an infectious MERS-CoV clone expressing the PREDICT/PDF-2180 spike protein was generated. Recombinant viruses derived from the clone were replication competent but unable to spread and establish new infections in Vero cells or primary human airway epithelial cells. Our findings suggest that PREDICT/PDF-2180 is unlikely to pose a zoonotic threat. Recombination in the S1 subunit of the spike gene was identified as the primary mechanism driving variation in the spike phenotype and was likely one of the critical steps in the evolution and emergence of MERS-CoV in humans.

## IMPORTANCE

Global surveillance efforts for undiscovered viruses are an important component of pandemic prevention initiatives. These surveys can be useful for finding novel viruses and for gaining insights into the ecological and evolutionary factors driving viral diversity; however, finding a viral sequence is not sufficient to determine whether it can infect people (i.e., poses a zoonotic threat). Here, we investigated the specific zoonotic risk of a MERS-like coronavirus (PREDICT/PDF-2180) identified in a bat from Uganda and showed that, despite being closely related to MERS-CoV, it is unlikely to pose a threat to humans. We suggest that this approach constitutes an appropriate strategy for beginning to determine the zoonotic potential of wildlife viruses. By showing that PREDICT/PDF-2180 does not infect cells that express the functional receptor for MERS-CoV, we further show that recombination was likely to be the critical step that allowed MERS to emerge in humans.

## INTRODUCTION

In 2012, Middle East respiratory syndrome (MERS) emerged in Saudi Arabia. Clusters of fatal pneumonia in adults were determined to be caused by a novel lineage C betacoronavirus (2c CoV), termed MERS-CoV (1). This was the first 2c CoV known to cause disease in humans and at the time of its discovery was most closely related to two known bat coronaviruses (2), raising the possibility that bats were a reservoir and source for the virus. Concurrently, epidemiologists identified an association between MERS infections in patients and their contact with dromedary camels (3, 4). MERS-CoV was subsequently detected in camels at a farm linked to two human cases in Qatar (5) and in camels in Egypt (6), followed by surveys that demonstrated widespread exposure to the virus in the Middle East and in North and East Africa as early as the 1980s (7–10). It is now clear that camels play an important role in the transmission of MERS-CoV to people (11), with seroprevalence highest among those who have had contact with camels (12).

While camels are thought to be important for the transmission of MERS-CoV, bats are widely considered to be the evolutionary source of the virus. Several 2c CoVs have now been described in bats, including HKU4 from *Tylonycteris pachypus* (13), HKU5 from *Pipistrellus abramus* (13), and the recently identified NeoCoV from *Neoromicia capensis* (14). NeoCoV is the closest relative yet discovered (85% identical to MERS) and shares sufficient genetic similarity in the replicase genes to be considered part of the same viral species (15); however, despite being closely related across much of the genome, the S1 subunit of the spike gene is highly divergent as a result of a prior recombination event. Recombination in the spike gene is particularly significant because the derived protein is responsible for host receptor recognition and membrane fusion (16) and thus is central in determining host specificity. The S1 subunit contains the receptor binding domain and therefore has a specific role in defining host tropism (17). Other processes are also important, such as the activation of the spike protein by host proteases (18), but the ability of S1 to bind with a host receptor is a critical step in the emergence pathway—and it can be quickly altered by a single recombination event. The sequence variation in the S1 region of MERS-CoV and NeoCoV could therefore indicate differences in host binding preferences.

Predicting the interactions of virus binding domains with a particular host receptor (for example, the human MERS-CoV receptor DPP4) is possible through the use of structural modeling and the generation of infectious clones. Protein-protein interactions can be modeled using a related homologous complex (19, 20) while reverse genetic strategies can test the permissiveness of human or other primate cells for infectious clones expressing the novel receptor binding domains or complete spike glycoprotein (21–24). Pseudotyped lentivirus systems have also been used, for example, to show that DPP4 is the receptor for HKU4 but not for the closely related HKU5 (25, 26). And while pseudotypes are not always accurate predictors of spike glycoprotein function (23), these findings indicate that multiple cell-entry strategies could exist for 2c viruses and that not all MERS-like CoVs pose an equal risk of zoonotic emergence.

Here, we investigated the receptor binding properties of a new strain of MERS-like CoV found in a bat from Uganda. This virus (PREDICT/PDF-2180) shares the same putative S1 subunit recombination that was observed in NeoCoV, allowing us to also consider whether the spike recombination was critical for the emergence of MERS-CoV in humans.

## RESULTS

### Sampling and site characterization.

**FIG 1:**
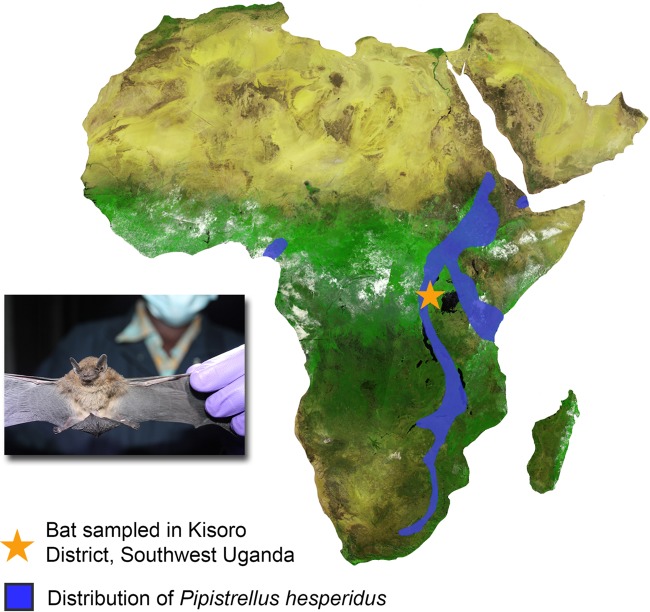
Map showing the distribution of *Pipistrellus hesperidus* (based on International Union for Conservation of Nature [IUCN] data) and the location of the bat sampled for the study.

A bat (identifier [ID] OTBA03-20130220) was trapped on 20 February 2013 in the Nkuringo area of Kisoro District, in southwestern Uganda (latitude −1.12, longitude 29.68) (Fig. 1). This area is an established settlement of villages comprising approximately 15,000 inhabitants adjacent to the southwestern boundary of Bwindi Impenetrable National Park. Communities include subsistence farmers growing small crops, with some members working inside the national park or supporting tourism-related businesses. Livestock, including cattle, pigs, sheep, goats, and poultry, are present in the village and are raised on a small scale primarily for local consumption.

The sampled bat weighed 3.0 g and had a forearm length of 25 mm (Fig. 1). It was identified as *Pipistrellus cf. hesperidus* based on 95% sequence identity in the cytochrome *b* (Cytb) gene. The cytochrome oxidase subunit 1 (CO1) was also sequenced, but no corresponding CO1 sequences for *P. hesperidus* were available in GenBank for comparison. We therefore relied on the Cytb sequence for species identification.

### Discovery and sequence characterization.

The oral swab, rectal swab, and whole blood of bat OTBA03-20130220 were assayed for the presence of coronavirus by consensus PCR (cPCR). Two separate assays were used, each targeting a different region of the ORF1b RNA-dependent RNA polymerase (RdRp). Bands of the expected size were amplified from the rectal swab (PDF-2180) by both assays and confirmed to represent viral products by traditional Sanger dideoxy sequencing. Both fragments showed >98% amino acid sequence identity to MERS-CoV, prompting further characterization of the virus. The oral swab and blood were negative.

The near-full-length genome (identified as PREDICT/PDF-2180) was assembled from 100-nucleotide (nt) Illumina single-end reads at an average depth of 26×. Only the 5′ and 3′ noncoding regions were left incomplete. The order of all predicted open reading frames (ORFs) was consistent with MERS-CoV and with the recently described NeoCoV (KC869678) identified in a bat from South Africa. Similarly, the hexanucleotide transcription regulatory sequence (AACGAA) was conserved and found in the same position as both MERS and NeoCoV upstream of each predicted ORF.

Across the full genome, the sequence had 86.5% amino acid identity to MERS-CoV and 91% to NeoCoV; however, considerable variation was observed in different genes. Amino acid identity could be as high as 97% to both MERS-CoV and NeoCoV in ORF1b or as low as 45% to MERS-CoV in subunit 1 of the spike protein. For the full spike protein, identity was 94% to NeoCoV and 63% to MERS-CoV. Percent sequence identity of the spike protein (subunits 1 and 2) to other 2c viruses is shown in Tables 1 and 2, respectively. Based on the current criteria for species demarcation established by the International Committee for the Taxonomy of Viruses (>90% amino acid sequence identity in the replicase proteins), PREDICT/PDF-2180 shares sufficient genetic identity to MERS-CoV to be considered a member of the *MERS-like Coronavirus* species.

**TABLE 1:** Pairwise amino acid sequence identity of subunit 1 of spike protein of 2c CoVs

Accession no. and/or isolate	% identity for subunit 1 (% identity for receptor binding domain)											
	NeoCoV	PDF-2180	EriCoV/2012/174	EriCoV/2012/216	BtCoV/133	HKU4	HKU5-1	HKU5-5	SC2013	EMC-2012	Al-Hasa1	NRC-HKU205
KC869678, NeoCoV												
Predict/PDF-2180	91.0 (93.1)											
KC545383, EriCoV/2012/174	54.7 (59.7)	54.3 (60.4)										
KC545386, EriCoV/2012/216	54.9 (59.7)	54.4 (60.4)	99.9 (100)									
DQ648794, BtCoV/133	45.1 (42.5)	45.5 (42.5)	43.9 (50.0)	44.0 (50.0)								
EF065505, BatCoV HKU4	45.1 (41.8)	45.7 (41.8)	44.3 (50.0)	44.4 (50.0)	95.6 (96.2)							
EF065509, BatCoV HKU5-1	47.8 (46.3)	47.7 (47.0)	45.8 (53.7)	45.9 (53.7)	57.7 (63.4)	58.4 (63.4)						
EF065512, BatCoV HKU5-5	47.4 (45.5)	47.8 (46.3)	44.6 (50.7)	44.7 (50.7)	56.3 (61.8)	56.7 (61.8)	90.1 (93.9)					
KJ473821, SC2013	45.5 (42.5)	46.2 (44.0)	45.8 (55.2)	45.9 (55.2)	59.7 (57.5)	59.7 (56.7)	63.7 (73.9)	64.1 (73.1)				
KC667074, EMC-2012	43.5 (40.3)	44.4 (41.0)	43.9 (47.8)	44.1 (47.8)	61.2 (64.9)	61.1 (63.4)	56.5 (63.4)	56.7 (61.1)	61.4 (61.2)			
KF186567, Al-Hasa1	43.5 (40.3)	44.4 (41.0)	43.9 (47.8)	44.1 (47.8)	61.4 (65.6)	61.2 (64.1)	56.5 (63.4)	56.7 (61.1)	61.4 (61.2)	99.9 (99.2)		
KJ477102, NRC-HKU205	43.7 (39.6)	44.4 (40.3)	43.8 (47.0)	43.9 (47.0)	61.1 (64.9)	61.0 (63.4)	56.3 (62.6)	56.4 (60.3)	61.4 (60.4)	99.1 (98.5)	99.2 (99.2)	

**TABLE 2:** Pairwise amino acid sequence identity of subunit 2 of spike protein of 2c CoVs

Accession no. and/or isolate	% identity											
	Al-Hasa1	EMC-2012	NRC-HKU205	NeoCoV	PDF-2180	EriCoV/2012/174	EriCoV/2012/216	BtCoV/133	HKU4	HKU5-1	HKU5-5	SC2013
KF186567, Al-Hasa1												
KC667074, EMC-2012	99.7											
KJ477102, NRC-HKU205	98.6	98.7										
KC869678, NeoCoV	84.7	84.9	84.4									
Predict/PDF-2180	85.5	85.7	84.9	97.6								
KC545383, EriCoV/2012/174	67.8	67.8	67.8	70.2	70.4							
KC545386, EriCoV/2012/216	68.3	68.3	68.3	70.5	70.7	96.9						
DQ648794, BtCoV/133	72.9	72.9	72.5	73.7	73.3	69.0	68.2					
EF065505, BatCoV HKU4	73.0	73.0	72.7	73.8	73.5	69.0	68.3	98.4				
EF065509, BatCoV HKU5-1	71.2	71.2	70.9	74.3	73.6	66.9	66.6	79.6	79.1			
EF065512, BatCoV HKU5-5	71.7	71.7	71.4	74.5	73.9	66.9	66.6	79.9	79.4	97.9		
KJ473821, SC2013	73.5	73.5	73.2	72.5	71.7	67.6	66.8	81.0	80.5	81.8	82.0	

### Phylogenetic analysis.

**FIG 2:**
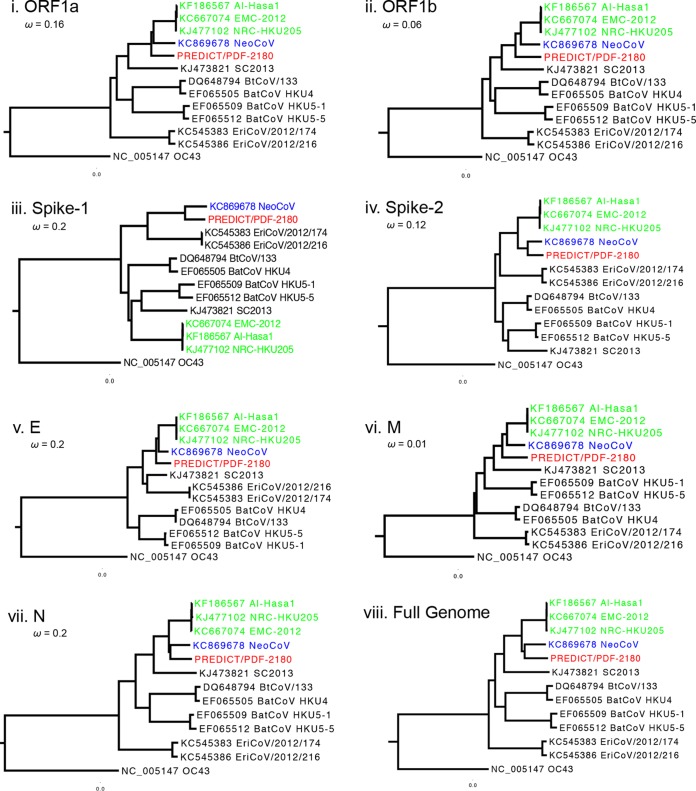
PREDICT/PDF-2180 and NeoCoV are ancestral to MERS-CoV. Maximum likelihood phylogenetic reconstructions of 2c coronaviruses (nucleotide) show that PREDICT/PDF-2180 and NeoCoV are consistently basal to, or form sister clades with, MERS-like CoV (human/camel strains), except in subunit 1 of the spike protein. Human OC43 is the outgroup. All genes were shown to be under purifying selection (ω).

Maximum likelihood phylogenetic reconstructions showed that PREDICT/PDF-2180 is most closely related to NeoCoV (Fig. 2). The two viruses were basal or formed sister clades to MERS-CoV in all genes except subunit 1 of the spike. The full-genome alignment was scanned for recombination using seven different algorithms (RDP, GENECONV, Bootscan, MaxChi, Chimaera, SiScan, and 3seq) implemented in RDP v4.46. A single recombination event was detected within the spike gene by RDP, Bootscan, MaxChi, Chimaera, SiScan, and 3seq (Bonferroni-corrected *P* of <<0.001), suggesting that the incongruent phylogenies observed between spike subunit 1 and the rest of the genome are the result of recombination. Attempts to date the divergence of these two viruses to estimate the “minimum” number of years since this recombination were prevented by evidence of strong negative (purifying) selection across the genome (Fig. 2). Given that purifying selection can confound true phylogenetic depth, we felt that attempts to estimate the number of years to common ancestry were inappropriate and would result in artificially “recent” dates.

### Zoonotic potential of PREDICT/PDF-2180.

**FIG 3:**
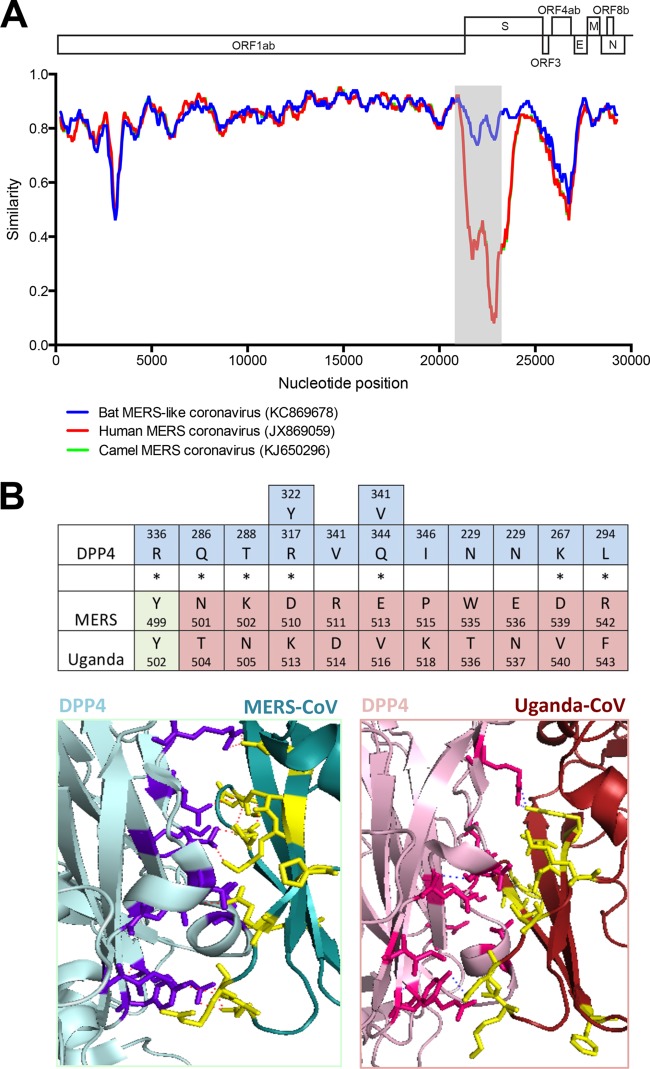
The spike protein of PREDICT/PDF-2180 is highly divergent. (A) A nucleotide identity Simplot shows that PREDICT/PDF-2180 and NeoCoV are closely related to MERS-CoV across much of the genome but are highly divergent in subunit 1 of the spike protein, suggesting that they may have different receptor binding properties. (B) Variation in key amino acid binding residues (*) and modeling to human DPP4 both suggest that PREDICT/PDF-2180 is unable to bind to DPP4.

The high genetic variability in subunit 1 suggests that human and bat strains of MERS have different receptor binding properties. To investigate this, we modeled the specific affinity of the PREDICT/PDF-2180 spike protein for the human MERS-CoV receptor DPP4 (27). We utilized the crystal structure of the MERS-CoV spike binding domain in complex with DPP4 to create a homology model for the comparable region of the PREDICT/PDF-2180 spike (Fig. 3). Previous work has demonstrated 11 specific amino acid residues in MERS-CoV that facilitate binding interactions with the human DPP4 (28). Of these residues, only one is conserved for PREDICT/PDF-2180. To determine whether the binding interactions may be conserved between DPP4 and PREDICT/PDF-2180 regardless of the differences in amino acid residues at these positions, we analyzed the predicted interactions between PREDICT/PDF-2180 and DPP4, compared to MERS-CoV and DPP4. Overall, we found a global reduction in predicted hydrogen bonding interactions in the DPP4-PREDICT/PDF-2180 binding interface compared with DPP4-MERS-CoV (Fig. 3). While the interactions in conserved residue Y499 were maintained, DPP4 interactions with PREDICT/PDF-2180 residues 501, 502, 510, 511, 513, 539, and 542 were disrupted. The interaction between DPP4 Y322 and MERS D510 is abolished in the PREDICT/PDF-2180 prediction, where D510 is replaced by K510. This is a charge change from negative to positive. Interestingly, a change from R511 in MERS to D511 in PREDICT/PDF-2180 facilitates a potential interaction with Y322 to replace the hydrogen bond lost with K510. Regardless, due to the predicted loss of the majority of the DPP4 binding interactions, the model predicts that PREDICT/PDF-2180 will not bind to DPP4.

**FIG 4:**
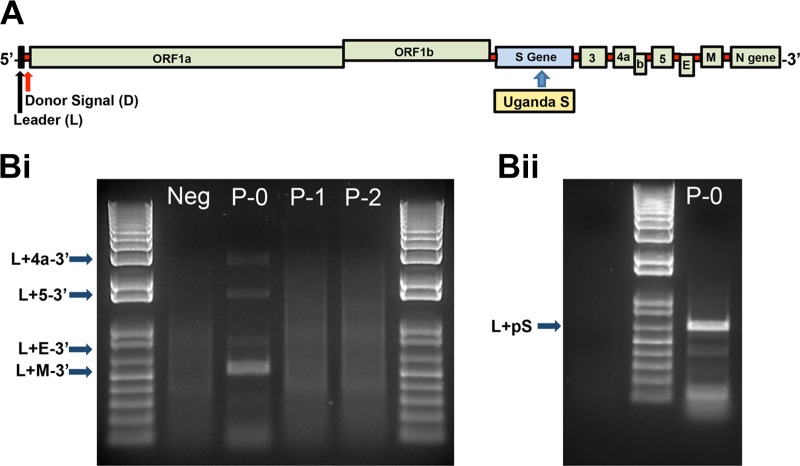
Uganda spike protein does not permit entry into Vero cells. (A) Genome organization of MERS-CoV encoding the Uganda spike glycoprotein. (Bi) Reverse transcription-PCR detection of leader-containing nested subgenomic mRNAs encoding the nucleocapsid transcript, E transcript, and ORF5 and ORF4a transcripts (p0, RNA-transfected cells; p1, passage 1; p2, passage 2). (Bii) Reverse transcription-PCR amplification of leader-containing mRNA 2 containing the Uganda S gene. Note the loss of the leader-containing transcripts in p1 and p2, demonstrating the loss of infectivity associated with insertion of the Uganda S gene. Ladder, 1 kb.

To confirm these results *in vitro*, a recombinant MERS-CoV cDNA clone was constructed containing the PREDICT/PDF-2180 spike gene in the context of the full-length MERS-CoV backbone. The chimeric virus maintains the entire ectodomain of the PREDICT/PDF-2180 spike with the exception of the first 20 amino acids of the 5′ end, which were taken from wild-type MERS-CoV. Similarly, the transmembrane domains (TMDs) and cytoplasmic tail of the chimeric virus used the wild-type MERS-CoV sequence in order to minimize incompatibility in virion formation. Following transfection into Vero cells, PCR amplification of leader-containing transcripts for all of the expected nested subgenomic (sg) mRNAs (including the sg spike mRNA) confirmed replication of the recombinant virus (Fig. 4). However, subsequent passages by supernatant transfer to uninfected monolayers failed to reproduce the infection, suggesting that the PREDICT/PDF-2180 spike protein is unable to mediate cell entry in Vero cells as seen with wild-type MERS-CoV (Fig. 4).

**FIG 5:**
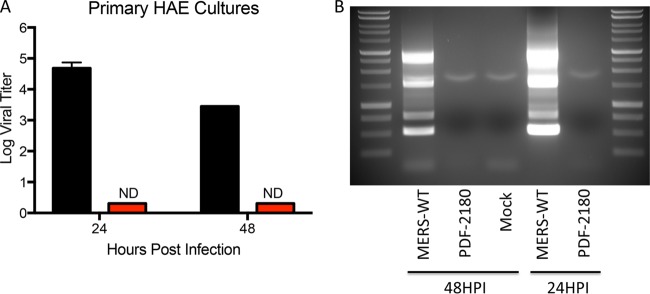
PDF-2180 spike unable to mediate infection of primary human airway cultures. (A) Primary human airway epithelial (HAE) cells grown on an air-liquid interface were infected with wild-type MERS-CoV (black bars) or passage 0 of PDF-2180/MERS chimeric CoV (red bars) and assayed by plaque assay on Vero cells. ND, none detected. (B) Reverse transcription-PCR detection of leader-containing nested subgenomic mRNAs encoding the nucleocapsid transcript, E transcript, and ORF5 and ORF4a transcripts following infection. Ladder, 1 kb; WT, wild type.

Supernatant from the transfected Vero cells (passage 0 [P0]) was also used to infect primary human airway epithelial (HAE) cells, which were derived from lung donors with no preexisting chronic disease. These well-differentiated primary cells are grown on an air-liquid interface and represent an important model for viral infection of the human lung. Several coronaviruses show improved replication in these polarized primary respiratory cells compared to standard cell lines. Using wild-type MERS-CoV as a control, primary HAE cell cultures were infected with passage 0 from the PREDICT/PDF-2180-MERS chimeric clone and showed no evidence of viral replication (Fig. 5A). Similarly, viral RNA expression analysis indicated no evidence of replication following infection with the PREDICT/PDF-2180 chimeric virus (Fig. 5B). In contrast, wild-type MERS-CoV induces robust replication as measured by plaque assay and viral-leader-containing transcripts. Together, the results indicate that the PREDICT/PDF-2180 spike is not likely to efficiently replicate in the human airway without further adaptation.

## DISCUSSION

The discovery of PREDICT/PDF-2180 in Uganda adds to the growing number of group C betacoronaviruses that have now been identified in bats. These include NeoCoV from South Africa (15), Mex_CoV-9 from Mexico (29), BatCoV/KW2E from Thailand (30), P.pipi/VM314 from the Netherlands (31), H.sav/206645-40 from Italy (32), and BetaCoV/SC2013, HKU4, and HKU5, all from China (33). Collectively, these examples demonstrate that the MERS-related CoVs are highly associated with bats and are geographically widespread.

The group 2c viruses appear to have a particular, though not exclusive, association with vespertilionid bats, which form a highly diverse and widely distributed family within the *Microchiroptera*. NeoCoV, SC2013, HKU4, HKU5, H.sav/206645-40, P.pipi/VM314, and PREDICT/PDF-2180 were all found in species belonging to this family. If the full diversity of 2c viruses reflects the number of vespertilionid species described (*n* = 475 species), there is potential for a substantial diversity of MERS-related viruses to be circulating in bats.

Our data suggest that PREDICT/PDF-2180 cannot infect humans and is not likely to pose a threat to human health, at least in its current form. The spike protein of this virus is distinct from the MERS-CoV spike, sharing only 46% amino acid identity, and it appears unable to enter cells that express the functional receptor used by MERS-CoV (DPP4)—or any other receptor expressed by either primate Vero cells or human airway epithelial cells. Importantly, failure to assemble and release viral particles from the initial infection could also explain our results; however, we suggest that receptor incompatibility is more likely given the steps taken to minimize particle disruption (see Materials and Methods). These results suggest that adaptation of the spike would be required to permit PREDICT/PDF-2180 replication in human airways. While we did not examine the specific binding properties of the related virus NeoCoV, the high amino acid sequence identity with PREDICT/PDF-2180 indicates that it shares a similar phenotype and is most likely also refractory for human infections.

Our data suggest that RNA recombination is the mechanism that underlies the observed difference in receptor binding. Recombination can occur at high frequency during mixed coronavirus infection, allowing different viral lineages to exchange specific functional motifs or even entire genes (22, 34, 35). Phylogenetic incongruence was noted in subunit 1 of the spike protein, and breakpoints were observed in this same region by multiple recombination detection algorithms. It is also parsimonious with the high purifying selection observed across the genome of 2c viruses (which argues against receptor adaptation via drift or selection) and with previous reports citing recombination in association with host switching for other coronaviruses (36–38). Given that the recombination is observed in both PREDICT/PDF-2180 and NeoCoV, we support the previous suggestion by Corman et al. (15) that it was the MERS-CoV that acquired a new spike. Given also that the PREDICT/PDF-2180 spike does not use DPP4 and is seemingly not competent for human infection, we further suggest that the recombination event was the critical factor driving the emergence of MERS-CoV.

What is less clear is whether this recombination occurred in bats or an intermediate host. Lineage 2c strains that use DPP4 have been reported in bats (25, 26), and there is also evidence of positive selection in the bat DPP4 that would indicate the existence of a large diversity of (as-yet-unknown) DPP4-competent strains (39). Just as detailed metagenomics studies have revealed the presence of several severe acute respiratory syndrome (SARS)-like bat CoVs that can use the human angiotensin converting enzyme 2 receptor and/or replicate efficiently in human cells (23, 24, 40–42), it seems likely that subsets of diverse MERS-CoV-like bat coronaviruses will also exist which are preprogrammed to efficiently use the human DPP4 receptor. This would support the hypothesis that the recombination occurred in bats; however, the MERS-CoV spike seems to have adapted and acquired a preference for human DPP4 over the bat homologue (26, 43) making it difficult to conclude with certainty that the MERS-CoV spike has bat origins. Increased surveillance will be required to understand the full diversity of spike phenotypes circulating in bats or in intermediate hosts such as camels.

In recent years, global surveillance efforts such as the USAID Emerging Pandemic Threats PREDICT program have advanced our understanding of the viral diversity that exists in wildlife (44). While this knowledge can be useful for proving the existence of novel viruses (29, 30, 45–49), quantifying overall viral diversity (45, 46), and measuring infection prevalence within a population, it does not provide information on their specific zoonotic threat. Given that no single correlate of pathogenicity or virulence has been determined for any viral family (50, 51) and that it is not possible to determine risk through phylogenetic data alone (51), the approach used here is an important tool in characterizing the zoonotic potential of viral sequences detected in wildlife. Doing so on a large scale (for example, as part of projects like USAID PREDICT) will also provide critical information on host and geographic variation in key viral traits, like potential host tropism, which are currently missing from most risk-based models forecasting hot spots of disease emergence.

## MATERIALS AND METHODS

### Sampling.

A bat (ID OTBA03-20130220) was trapped on 20 February 2013 in the Nkuringo area of Kisoro District in southwestern Uganda. The bat was caught with a mist net (3.8-mm mesh; Avinet, Inc.) according to established protocols and was released unharmed postsampling. Standard morphometric measurements (weight and forearm length) and photographs were obtained to aid species identification, which was confirmed by DNA barcoding of the cytochrome *b* (Cytb) and cytochrome oxidase subunit 1 (CO1) mitochondrial DNA genes (52). Approximately 200 µl of whole blood was collected into EDTA. Oral and rectal swabs were also collected in duplicate (one into viral transport medium and one dry). Specimens were stored temporarily on gel packs and frozen in liquid nitrogen in the field within 4 h of collection and then transferred to −80°C for storage until testing. Samples were transferred to the Center for Infection and Immunity at Columbia University for viral discovery and characterization.

### Coronavirus discovery by consensus PCR.

Total nucleic acid (TNA) was extracted using the Roche MagNA Pure 96 platform according to the manufacturer’s instructions. TNA was reverse transcribed into cDNA using SuperScript III (Invitrogen) according to the manufacturer’s instructions. Two broadly reactive consensus PCR assays targeting partial and nonoverlapping regions of the coronavirus ORF1b (containing the RdRp) were performed (53, 54). Bands of the expected size were excised from 1% agarose, cloned into Strataclone PCR cloning vector, and sequenced to confirm detection.

### Sequencing and bioinformatic processing.

Total RNA extract was DNase treated (DNase I; Ambion, Life Technologies, Inc.) and reverse transcribed using SuperScript III (Invitrogen, Life Technologies, Inc.) with random hexamer primers. The cDNA was RNase H treated before second-strand synthesis with Klenow fragment (3′ to 5′ exonuclease) (New England Biolabs). The resulting double-stranded cDNA was sheared to 200-bp (average) fragments using a Covaris focused ultrasonicator E210, according to the manufacturer’s standard settings, and used for library construction using the Kapa Hyper library preparation kit (Kapa Biosystems, Roche), again according to the manufacturer’s instructions. The final library was quantified using an Agilent Bioanalyzer 2100 and pooled to allocate 20 million reads on the Illumina HiSeq 2500 platform.

The Q30-filtered FastQ files were used to generate quality control reports using PRINSEQ software (v0.20.2) (55) and were further filtered and trimmed. Host background levels were determined by mapping the filtered reads against a bat reference database using Bowtie2 mapper (v2.0.6, http://bowtie-bio.sourceforge.net) (56). The host-subtracted reads were *de novo* assembled using MIRA assembler (v4.0) (57). Contigs and unique singletons were subjected to homology search using MegaBlast against the GenBank nucleotide database. Sequences that showed poor or no homology at the nucleotide level were screened by BLASTX against the viral GenBank protein database. Viral sequences from BLASTX analysis were subjected to another round of BLASTX homology search against the entire GenBank protein database to correct for biased E values and taxonomic misassignments. The genome of PREDICT/PDF-2180 was mapped with Bowtie2 against the filtered data set to visualize depth and coverage in Integrated Genomics Viewer.

### Genetic and phylogenetic analyses.

Sequences were analyzed and edited using Geneious (version 6.0.3). Full genome and individual gene sequences were aligned with ClustalW, and maximum likelihood phylogenetic trees were constructed in PAUP* (500 bootstraps). Models of nucleotide substitution were selected using jModelTest. Nucleotide sequence similarity between MERS-like viruses was assessed using Simplot v3.5.1 (58) with a sliding window size of 500 bp, a step size of 50 nucleotides, and 1,000 bootstrap replicates using gap-stripped alignments and the F84 (maximum likelihood) distance model. The full-genome alignment was scanned for recombination using seven different algorithms (RDP, GENECONV, Bootscan, MaxChi, Chimaera, SiScan, and 3seq) implemented in RDP (v4.46) (59).

### Structural modeling.

Predicted binding differences between DPP4 and either MERS or Uganda were determined by structural analysis. The crystal structure demonstrating the interactions between DPP4 and MERS spike binding domain has previously been reported (28), and the crystal structure is PDB ID 4KR0. We created a homology model of the region of the Uganda spike protein homologous to the MERS spike binding domain based on the 4KR0 structure in association with DPP4. We first aligned the amino acid sequences for 4KR0 (28) and the Uganda spike using Clustal Omega (60). We then used MODELLER (61) to create predicted structural coordinates for the Uganda spike based on the coordinates of 4KR0. Because MODELLER requires the two sequences to be the same length, we introduced gaps in the sequences where appropriate to maintain the best sequence identity between the 2 amino acid sequences. Numbering is based on MERS-CoV amino acid residues. We then imported the predicted crystal structure for Uganda and the known DPP4-MERS structure into PyMOL (62) for visualization and comparative analysis. Hydrogen bonding interactions were predicted by selecting the known DPP4 and 4KR0 or the homologous DPP4 and Uganda interaction sites and using the “find polar interactions” function within PyMOL.

### Generation of a MERS-CoV recombinant virus.

Previously, we reported the isolation of recombinant MERS-CoV that was derived from a cDNA clone (63). To reconstitute a MERS genome expressing the PREDICT/PDF-2180 CoV spike, new E and F plasmids were ordered synthetically (Bio-Basic) to contain the PREDICT/PDF-2180 spike ectodomain; these plasmids were named MERS-Uganda E and F. MERS ORF1 and ORF2 overlap, so to maintain a functional replicase sequence and signal sequence for spike, the first 20 amino acids of the MERS spike were retained and the PREDICT/PDF-2180 sequence was fused in frame downstream of the MERS-CoV S glycoprotein signal peptidase domain beginning at its 24th amino acid. In short, the sequence of the MERS spike coding for amino acids 21 to 1306 was replaced with the sequence of the PREDICT/PDF-2180 spike coding for amino acids 24 to 1298, so that following processing, an intact spike glycoprotein was expressed during virus infection. The E and F plasmids were sequence verified prior to the assembly of full-length recombinant DNAs.

The MERS A through F inserts (containing the Uganda S gene) were restriction digested, resolved on 0.8% agarose gels, visualized, excised, and purified using a QIAquick gel extraction kit (Qiagen). The MERS A to F inserts were mixed and ligated overnight at 4°C, phenol-chloroform extracted, and precipitated under isopropyl alcohol. Full-length T7 transcripts were generated *in vitro* as described by the manufacturer (Ambion; mMessage mMachine) with certain modifications (63). For MERS-CoV N transcripts, 1 μg of plasmid DNA containing the N gene (amplified using forward primer 5′-ATTTAGGTGACACTATAGATGGCATCCCCTGCTGCACC-3′ and reverse primer 5′-TTTTTTTTTTTTTTTTTTTTTTCTAATCAGTGTTAACATCAATCATTGG-3′) was transcribed by SP6 RNA polymerase with a 4:1 ratio of cap analog to GTP. RNA transcripts were added to 800 μl of Vero cell suspension (8.0 × 10^6^ cells) in an electroporation cuvette, and four electrical pulses of 450 V at 50 μF were delivered with a Gene Pulser II electroporator (Bio-Rad). The transfected Vero cells were allowed to recover for 10 min at room temperature and then incubated at 37°C for 2 to 4 days in a 75-cm^2^ flask. Virus progeny were then passaged several times in Vero cells or primary human airway epithelial cells for 48 h to detect viable viruses. All viruses were maintained under biosafety level 3 (BSL3) conditions with redundant fans, and personnel used powered air-purifying respirators (PAPRs) and Tyvek suits.

To detect leader-containing RNAs, intracellular RNA from wild type and recombinant MERS-CoV-Uganda (rMERS-CoV-Uganda) was reverse transcribed with a primer at the 3′ end of the genome and cDNA was isolated for PCR using a reverse primer located in ORF5 and a forward primer located in the leader RNA sequence at the 5′ end of the genome (5′-CTATCTCACTTCCCCTCGTTCTC-3′). Leader-containing amplicons were sequenced as previously described (64). The cDNA products were separated and visualized in 0.8% agarose gels.

### Viruses, cells, and infection.

Wild-type and chimeric CoVs were cultured on Vero E6 cells, grown in Dulbecco modified Eagle medium (DMEM) (Gibco, Carlsbad, CA) and 5% fetal clone serum (HyClone, South Logan, UT) along with antibiotic-antimycotic (anti-anti; Gibco, Carlsbad, CA). Growth curves in Vero and primary human airway epithelial cells were performed as previously described (65, 66). Human lungs were procured under University of North Carolina at Chapel Hill Institutional Review Board-approved protocols.

### Biosafety and biosecurity.

Reported studies were initiated after the NIH and the University of North Carolina Institutional Biosafety Committee approved the experimental protocol (project title, Generating Infectious Clones of Bat SARS-like CoVs; lab safety plan ID, 20167715; schedule G ID, 19982).

### Accession number(s).

The near-complete genome sequence for PREDICT/PDF-2180 has been deposited in GenBank under accession number KX574227.

## References

[1] Zaki AM, van Boheemen S, Bestebroer TM, Osterhaus AD, Fouchier RA 2012 Isolation of a novel coronavirus from a man with pneumonia in Saudi Arabia. N Engl J Med 367:1814–1820. doi:10.1056/NEJMoa1211721. 23075143

[2] Woo PC, Lau SK, Li KS, Tsang AK, Yuen KY 2012 Genetic relatedness of the novel human group C betacoronavirus to Tylonycteris bat coronavirus HKU4 and Pipistrellus bat coronavirus HKU5. Emerg Microbes Infect 1:e35. doi:10.1038/emi.2012.45. 26038405 PMC3630921

[3] Reusken CB, Haagmans BL, Müller MA, Gutierrez C, Godeke GJ, Meyer B, Muth D, Raj VS, Smits-De Vries L, Corman VM, Drexler JF, Smits SL, El Tahir YE, De Sousa R, van Beek J, Nowotny N, van Maanen K, Hidalgo-Hermoso E, Bosch BJ, Rottier P, Osterhaus A, Gortázar-Schmidt C, Drosten C, Koopmans MP 2013 Middle East respiratory syndrome coronavirus neutralising serum antibodies in dromedary camels: a comparative serological study. Lancet Infect Dis 13:859–866. doi:10.1016/S1473-3099(13)70164-6. 23933067 PMC7106530

[4] Azhar EI, El-Kafrawy SA, Farraj SA, Hassan AM, Al-Saeed MS, Hashem AM, Madani TA 2014 Evidence for camel-to-human transmission of MERS coronavirus. N Engl J Med 370:2499–2505. doi:10.1056/NEJMoa1401505. 24896817

[5] Haagmans BL, Al Dhahiry SH, Reusken CB, Raj VS, Galiano M, Myers R, Godeke GJ, Jonges M, Farag E, Diab A, Ghobashy H, Alhajri F, Al-Thani M, Al-Marri SA, Al Romaihi HE, Al Khal A, Bermingham A, Osterhaus AD, AlHajri MM, Koopmans MP 2014 Middle East respiratory syndrome coronavirus in dromedary camels: an outbreak investigation. Lancet Infect Dis 14:140–145. doi:10.1016/S1473-3099(13)70690-X. 24355866 PMC7106553

[6] Chu DK, Poon LL, Gomaa MM, Shehata MM, Perera RA, Abu Zeid D, El Rifay AS, Siu LY, Guan Y, Webby RJ, Ali MA, Peiris M, Kayali G 2014 MERS coronaviruses in dromedary camels, Egypt. Emerg Infect Dis 20:1049–1053. doi:10.3201/eid2006.140299. 24856660 PMC4036765

[7] Reusken CB, Messadi L, Feyisa A, Ularamu H, Godeke GJ, Danmarwa A, Dawo F, Jemli M, Melaku S, Shamaki D, Woma Y, Wungak Y, Gebremedhin EZ, Zutt I, Bosch BJ, Haagmans BL, Koopmans MP 2014 Geographic distribution of MERS coronavirus among dromedary camels, Africa. Emerg Infect Dis 20:1370–1374. doi:10.3201/eid2008.140590. 25062254 PMC4111168

[8] Corman VM, Jores J, Meyer B, Younan M, Liljander A, Said MY, Gluecks I, Lattwein E, Bosch BJ, Drexler JF, Bornstein S, Drosten C, Müller MA 2014 Antibodies against MERS coronavirus in dromedary camels, Kenya, 1992–2013. Emerg Infect Dis 20:1319–1322. doi:10.3201/eid2008.140596. 25075637 PMC4111164

[9] Müller MA, Corman VM, Jores J, Meyer B, Younan M, Liljander A, Bosch BJ, Lattwein E, Hilali M, Musa BE, Bornstein S, Drosten C 2014 MERS coronavirus neutralizing antibodies in camels, eastern Africa, 1983–1997. Emerg Infect Dis 20:2093–2095. doi:10.3201/eid2012.141026. 25425139 PMC4257824

[10] Alagaili AN, Briese T, Mishra N, Kapoor V, Sameroff SC, Burbelo PD, de Wit E, Munster VJ, Hensley LE, Zalmout IS, Kapoor A, Epstein JH, Karesh WB, Daszak P, Mohammed OB, Lipkin WI 2014 Middle East respiratory syndrome coronavirus infection in dromedary camels in Saudi Arabia. mBio 5:e00884-14. doi:10.1128/mBio.00884-14. 24570370 PMC3940034

[11] Azhar EI, Hashem AM, El-Kafrawy SA, Sohrab SS, Aburizaiza AS, Farraj SA, Hassan AM, Al-Saeed MS, Jamjoom GA, Madani TA 2014 Detection of the Middle East respiratory syndrome coronavirus genome in an air sample originating from a camel barn owned by an infected patient. mBio 5:e01450-14. doi:10.1128/mBio.01450-14. 25053787 PMC4120199

[12] Müller MA, Meyer B, Corman VM, Al-Masri M, Turkestani A, Ritz D, Sieberg A, Aldabbagh S, Bosch B, Lattwein E, Alhakeem RF, Assiri AM, Albarrak AM, Al-Shangiti AM, Al-Tawfiq JA, Wikramaratna P, Alrabeeah AA, Drosten C, Memish ZA 2015 Presence of Middle East respiratory syndrome coronavirus antibodies in Saudi Arabia: a nationwide, cross-sectional, serological study. Lancet Infect Dis 15:559–564. doi:10.1016/S1473-3099(15)70090-3. 25863564 PMC7185864

[13] Woo PC, Lau SK, Li KS, Poon RW, Wong BH, Tsoi HW, Yip BC, Huang Y, Chan KH, Yuen KY 2006 Molecular diversity of coronaviruses in bats. Virology 351:180–187. doi:10.1016/j.virol.2006.02.041. 16647731 PMC7111821

[14] Ithete NL, Stoffberg S, Corman VM, Cottontail VM, Richards LR, Schoeman MC, Drosten C, Drexler JF, Preiser W 2013 Close relative of human Middle East respiratory syndrome coronavirus in bat, South Africa. Emerg Infect Dis 19:1697–1699. doi:10.3201/eid1910.130946. 24050621 PMC3810765

[15] Corman VM, Ithete NL, Richards LR, Schoeman MC, Preiser W, Drosten C, Drexler JF 2014 Rooting the phylogenetic tree of Middle East respiratory syndrome coronavirus by characterization of a conspecific virus from an African bat. J Virol 88:11297–11303. doi:10.1128/JVI.01498-14. 25031349 PMC4178802

[16] Gallagher TM, Buchmeier MJ 2001 Coronavirus spike proteins in viral entry and pathogenesis. Virology 279:371–374. doi:10.1006/viro.2000.0757. 11162792 PMC7133764

[17] Lu G, Wang Q, Gao GF 2015 Bat-to-human: spike features determining “host jump” of coronaviruses SARS-CoV, MERS-CoV, and beyond. Trends Microbiol 23:468–478. doi:10.1016/j.tim.2015.06.003. 26206723 PMC7125587

[18] Millet JK, Whittaker GR 2015 Host cell proteases: critical determinants of coronavirus tropism and pathogenesis. Virus Res 202:120–134. doi:10.1016/j.virusres.2014.11.021. 25445340 PMC4465284

[19] Martí-Renom MA, Stuart AC, Fiser A, Sánchez R, Melo F, Sali A 2000 Comparative protein structure modeling of genes and genomes. Annu Rev Biophys Biomol Struct 29:291–325. doi:10.1146/annurev.biophys.29.1.291. 10940251

[20] Szilagyi A, Zhang Y 2014 Template-based structure modeling of protein-protein interactions. Curr Opin Struct Biol 24:10–23. doi:10.1016/j.sbi.2013.11.005. 24721449 PMC3984454

[21] Rockx B, Sheahan T, Donaldson E, Harkema J, Sims A, Heise M, Pickles R, Cameron M, Kelvin D, Baric R 2007 Synthetic reconstruction of zoonotic and early human severe acute respiratory syndrome coronavirus isolates that produce fatal disease in aged mice. J Virol 81:7410–7423. doi:10.1128/JVI.00505-07. 17507479 PMC1933338

[22] Becker MM, Graham RL, Donaldson EF, Rockx B, Sims AC, Sheahan T, Pickles RJ, Corti D, Johnston RE, Baric RS, Denison MR 2008 Synthetic recombinant bat SARS-like coronavirus is infectious in cultured cells and in mice. Proc Natl Acad Sci U S A 105:19944–19949. doi:10.1073/pnas.0808116105. 19036930 PMC2588415

[23] Menachery VD, Yount BL, Debbink K, Agnihothram S, Gralinski LE, Plante JA, Graham RL, Scobey T, Ge XY, Donaldson EF, Randell SH, Lanzavecchia A, Marasco WA, Shi ZL, Baric RS 2015 A SARS-like cluster of circulating bat coronaviruses shows potential for human emergence. Nat Med 21:1508–1513. doi:10.1038/nm.3985. 26552008 PMC4797993

[24] Menachery VD, Yount BL, Sims AC, Debbink K, Agnihothram SS, Gralinski LE, Graham RL, Scobey T, Plante JA, Royal SR, Swanstrom J, Sheahan TP, Pickles RJ, Corti D, Randell SH, Lanzavecchia A, Marasco WA, Baric RS 2016 SARS-like WIV1-CoV poised for human emergence. Proc Natl Acad Sci U S A 113:3048–3053. doi:10.1073/pnas.1517719113. 26976607 PMC4801244

[25] Wang Q, Qi J, Yuan Y, Xuan Y, Han P, Wan Y, Ji W, Li Y, Wu Y, Wang J, Iwamoto A, Woo PC, Yuen KY, Yan J, Lu G, Gao GF 2014 Bat origins of MERS-CoV supported by bat coronavirus HKU4 usage of human receptor CD26. Cell Host Microbe 16:328–337. doi:10.1016/j.chom.2014.08.009. 25211075 PMC7104937

[26] Yang Y, Du L, Liu C, Wang L, Ma C, Tang J, Baric RS, Jiang S, Li F 2014 Receptor usage and cell entry of bat coronavirus HKU4 provide insight into bat-to-human transmission of MERS coronavirus. Proc Natl Acad Sci U S A 111:12516–12521. doi:10.1073/pnas.1405889111. 25114257 PMC4151778

[27] Raj VS, Mou H, Smits SL, Dekkers DHW, Müller MA, Dijkman R, Muth D, Demmers JAA, Zaki A, Fouchier RAM, Thiel V, Drosten C, Rottier PJM, Osterhaus ADME, Bosch BJ, Haagmans BL 2013 Dipeptidyl peptidase 4 is a functional receptor for the emerging human coronavirus-EMC. Nature 495:251–254. doi:10.1038/nature12005. 23486063 PMC7095326

[28] Lu G, Hu Y, Wang Q, Qi J, Gao F, Li Y, Zhang Y, Zhang W, Yuan Y, Bao J, Zhang B, Shi Y, Yan J, Gao GF 2013 Molecular basis of binding between novel human coronavirus MERS-CoV and its receptor CD26. Nature 500:227–231. doi:10.1038/nature12328. 23831647 PMC7095341

[29] Anthony SJ, Ojeda-Flores R, Rico-Chávez O, Navarrete-Macias I, Zambrana-Torrelio CM, Rostal MK, Epstein JH, Tipps T, Liang E, Sanchez-Leon M, Sotomayor-Bonilla J, Aguirre AA, Ávila-Flores R, Medellin RA, Goldstein T, Suzán G, Daszak P, Lipkin WI 2013 Coronaviruses in bats from Mexico. J Gen Virol 94:1028–1038. doi:10.1099/vir.0.049759-0. 23364191 PMC3709589

[30] Wacharapluesadee S, Sintunawa C, Kaewpom T, Khongnomnan K, Olival KJ, Epstein JH, Rodpan A, Sangsri P, Intarut N, Chindamporn A, Suksawa K, Hemachudha T 2013 Group C betacoronavirus in bat guano fertilizer, Thailand. Emerg Infect Dis 19:1349–1351. doi:10.3201/eid1908.130119. 23880503 PMC3739538

[31] Reusken CBEM, Lina PHC, Pielaat A, de Vries A, Dam-Deisz C, Adema J, Drexler JF, Drosten C, Kooi EA 2010 Circulation of group 2 coronaviruses in a bat species common to urban areas in Western Europe. Vector Borne Zoonotic Dis 10:785–791. doi:10.1089/vbz.2009.0173. 20055576

[32] Lelli D, Papetti A, Sabelli C, Rosti E, Moreno A, Boniotti MB 2013 Detection of coronaviruses in bats of various species in Italy. Viruses 5:2679–2689. doi:10.3390/v5112679. 24184965 PMC3856409

[33] Woo PCY, Wang M, Lau SK, Xu H, Poon RW, Guo R, Wong BH, Gao K, Tsoi HW, Huang Y, Li KS, Lam CS, Chan KH, Zheng BJ, Yuen KY 2007 Comparative analysis of twelve genomes of three novel group 2c and group 2d coronaviruses reveals unique group and subgroup features. J Virol 81:1574–1585. doi:10.1128/JVI.02182-06. 17121802 PMC1797546

[34] Baric RS, Fu K, Schaad MC, Stohlman SA 1990 Establishing a genetic recombination map for murine coronavirus strain A59 complementation groups. Virology 177:646–656. doi:10.1016/0042-6822(90)90530-5. 2164728 PMC7130460

[35] Banner LR, Keck JG, Lai MM 1990 A clustering of RNA recombination sites adjacent to a hypervariable region of the peplomer gene of murine coronavirus. Virology 175:548–555. doi:10.1016/0042-6822(90)90439-X. 2158184 PMC7130556

[36] Du J, Yang L, Ren X, Zhang J, Dong J, Sun L, Zhu Y, Yang F, Zhang S, Wu Z, Jin Q 2016 Genetic diversity of coronaviruses in Miniopterus fuliginosus bats. Sci China Life Sci 59:604–614. doi:10.1007/s11427-016-5039-0. 27125516 PMC7089092

[37] Lau SK, Feng Y, Chen H, Luk HK, Yang WH, Li KS, Zhang YZ, Huang Y, Song ZZ, Chow WN, Fan RY, Ahmed SS, Yeung HC, Lam CS, Cai JP, Wong SS, Chan JF, Yuen KY, Zhang HL, Woo PC 2015 Severe acute respiratory syndrome (SARS) coronavirus ORF8 protein is acquired from SARS-related coronavirus from greater horseshoe bats through recombination. J Virol 89:10532–10547. doi:10.1128/JVI.01048-15. 26269185 PMC4580176

[38] Woo PC, Lau SK, Huang Y, Yuen KY 2009 Coronavirus diversity, phylogeny and interspecies jumping. Exp Biol Med (Maywood) 234:1117–1127. doi:10.3181/0903-MR-94. 19546349

[39] Cui J, Eden JS, Holmes EC, Wang LF 2013 Adaptive evolution of bat dipeptidyl peptidase 4 (DPP4): implications for the origin and emergence of Middle East respiratory syndrome coronavirus. Virol J 10:304. doi:10.1186/1743-422X-10-304. 24107353 PMC3852826

[40] Zeng LP, Gao YT, Ge XY, Zhang Q, Peng C, Yang XL, Tan B, Chen J, Chmura AA, Daszak P, Shi ZL 2016 Bat severe acute respiratory syndrome-like coronavirus WIV1 encodes an extra accessory protein, ORFX, involved in modulation of the host immune response. J Virol 90:6573–6582. doi:10.1128/JVI.03079-15. 27170748 PMC4936131

[41] Yang XL, Hu B, Wang B, Wang MN, Zhang Q, Zhang W, Wu LJ, Ge XY, Zhang YZ, Daszak P, Wang LF, Shi ZL 2015 Isolation and characterization of a novel bat coronavirus closely related to the direct progenitor of severe acute respiratory syndrome coronavirus. J Virol 90:3253–3256. doi:10.1128/JVI.02582-15. 26719272 PMC4810638

[42] Ge XY, Li JL, Yang XL, Chmura AA, Zhu G, Epstein JH, Mazet JK, Hu B, Zhang W, Peng C, Zhang YJ, Luo CM, Tan B, Wang N, Zhu Y, Crameri G, Zhang SY, Wang LF, Daszak P, Shi ZL 2013 Isolation and characterization of a bat SARS-like coronavirus that uses the ACE2 receptor. Nature 503:535–538. doi:10.1038/nature12711. 24172901 PMC5389864

[43] Barlan A, Zhao J, Sarkar MK, Li K, McCray PB, Perlman S, Gallagher T 2014 Receptor variation and susceptibility to Middle East respiratory syndrome coronavirus infection. J Virol 88:4953–4961. doi:10.1128/JVI.00161-14. 24554656 PMC3993797

[44] PREDICT Consortium. 2014 Reducing pandemic risk, promoting global health. One Health Institute, University of California, Davis, CA.

[45] Anthony SJ, Epstein JH, Murray KA, Navarrete-Macias I, Zambrana-Torrelio CM, Solovyov A, Ojeda-Flores R, Arrigo NC, Islam A, Ali Khan S, Hosseini P, Bogich TL, Olival KJ, Sanchez-Leon MD, Karesh WB, Goldstein T, Luby SP, Morse SS, Mazet JA, Daszak P, Lipkin WI 2013 A strategy to estimate unknown viral diversity in mammals. mBio 4:e00598-13. doi:10.1128/mBio.00598-13. 24003179 PMC3760253

[46] Anthony SJ, Islam A, Johnson C, Navarrete-Macias I, Liang E, Jain K, Hitchens PL, Che X, Soloyvov A, Hicks AL, Ojeda-Flores R, Zambrana-Torrelio C, Ulrich W, Rostal MK, Petrosov A, Garcia J, Haider N, Wolfe N, Goldstein T, Morse SS, Rahman M, Epstein JH, Mazet JK, Daszak P, Lipkin WI 2015 Non-random patterns in viral diversity. Nat Commun 6:8147. doi:10.1038/ncomms9147. 26391192 PMC4595600

[47] Wacharapluesadee S, Duengkae P, Rodpan A, Kaewpom T, Maneeorn P, Kanchanasaka B, Yingsakmongkon S, Sittidetboripat N, Chareesaen C, Khlangsap N, Pidthong A, Leadprathom K, Ghai S, Epstein JH, Daszak P, Olival KJ, Blair PJ, Callahan MV, Hemachudha T 2015 Diversity of coronavirus in bats from Eastern Thailand. Virol J 12:57. doi:10.1186/s12985-015-0289-1. 25884446 PMC4416284

[48] Seimon TA, Olson SH, Lee KJ, Rosen G, Ondzie A, Cameron K, Reed P, Anthony SJ, Joly DO, Karesh WB, McAloose D, Lipkin WI 2015 Adenovirus and herpesvirus diversity in free-ranging great apes in the Sangha region of the Republic of Congo. PLoS One 10:e0118543. doi:10.1371/journal.pone.0118543. 25781992 PMC4362762

[49] Hu B, Chmura AA, Li J, Zhu G, Desmond JS, Zhang Y, Zhang W, Epstein JH, Daszak P, Shi Z 2014 Detection of diverse novel astroviruses from small mammals in China. J Gen Virol 95:2442–2449. doi:10.1099/vir.0.067686-0. 25034867

[50] Daszak P, Lipkin WI 2011 The search for meaning in virus discovery. Curr Opin Virol 1:620–623. doi:10.1016/j.coviro.2011.10.010. 22440920 PMC4310690

[51] Morse SS, Mazet JA, Woolhouse M, Parrish CR, Carroll D, Karesh WB, Zambrana-Torrelio C, Lipkin WI, Daszak P 2012 Prediction and prevention of the next pandemic zoonosis. Lancet 380:1956–1965. doi:10.1016/S0140-6736(12)61684-5. 23200504 PMC3712877

[52] Townzen JS, Brower AV, Judd DD 2008 Identification of mosquito bloodmeals using mitochondrial cytochrome oxidase subunit I and cytochrome *b* gene sequences. Med Vet Entomol 22:386–393. doi:10.1111/j.1365-2915.2008.00760.x. 19120966

[53] Quan P-L, Firth C, Street C, Henriquez JA, Petrosov A, Tashmukhamedova A, Hutchison SK, Egholm M, Osinubi MOV, Niezgoda M, Ogunkoya AB, Briese T, Rupprecht CE, Lipkin WI 2010 Identification of a severe acute respiratory syndrome coronavirus-like virus in a leaf-nosed bat in Nigeria. mBio 1:e00208-10. doi:10.1128/mBio.00208-10. 21063474 PMC2975989

[54] Watanabe S, Masangkay JS, Nagata N, Morikawa S, Mizutani T, Fukushi S, Alviola P, Omatsu T, Ueda N, Iha K, Taniguchi S, Fujii H, Tsuda S, Endoh M, Kato K, Tohya Y, Kyuwa S, Yoshikawa Y, Akashi H 2010 Bat coronaviruses and experimental infection of bats, the Philippines. Emerg Infect Dis 16:1217–1223. doi:10.3201/Eid1608.100208. 20678314 PMC3298303

[55] Schmieder R, Edwards R 2011 Quality control and preprocessing of metagenomic datasets. Bioinformatics 27:863–864. doi:10.1093/bioinformatics/btr026. 21278185 PMC3051327

[56] Langmead B, Salzberg SL 2012 Fast gapped-read alignment with bowtie 2. Nat Methods 9:357–359. doi:10.1038/nmeth.1923. 22388286 PMC3322381

[57] Chevreux B, Wetter T, Suhai S 1999 Genome sequence assembly using trace signals and additional sequence information, p 45–56. *In* Wingender E (ed), Computer science and biology: proceedings of the German Conference on Bioinformatics, GCB '99; October 4–6, 1999, Hannover, Germany.

[58] Lole KS, Bollinger RC, Paranjape RS, Gadkari D, Kulkarni SS, Novak NG, Ingersoll R, Sheppard HW, Ray SC 1999 Full-length human immunodeficiency virus type 1 genomes from subtype C-infected seroconverters in India, with evidence of intersubtype recombination. J Virol 73:152–160. 9847317 10.1128/jvi.73.1.152-160.1999 PMC103818

[59] Martin DP, Murrell B, Golden M, Khoosal A, Muhire B 2015 RDP4: detection and analysis of recombination patterns in virus genomes. Virus Evol 1:vev003. doi:10.1093/ve/vev003. 27774277 PMC5014473

[60] Sievers F, Wilm A, Dineen D, Gibson TJ, Karplus K, Li W, Lopez R, McWilliam H, Remmert M, Söding J, Thompson JD, Higgins DG 2011 Fast, scalable generation of high-quality protein multiple sequence alignments using Clustal Omega. Mol Syst Biol 7:539. doi:10.1038/msb.2011.75. 21988835 PMC3261699

[61] Webb B, Sali A 2014 Comparative protein structure modeling using MODELLER. Curr Protoc Bioinformatics 47:5.6.1–5.6.32. doi:10.1002/0471250953.bi0506s47. 25199792

[62] Schrödinger LLC 2015 The PyMOL molecular graphics system, version 1.8. Schrödinger LLC, New York, NY.

[63] Scobey T, Yount BL, Sims AC, Donaldson EF, Agnihothram SS, Menachery VD, Graham RL, Swanstrom J, Bove PF, Kim JD, Grego S, Randell SH, Baric RS 2013 Reverse genetics with a full-length infectious cDNA of the Middle East respiratory syndrome coronavirus. Proc Natl Acad Sci U S A 110:16157–16162. doi:10.1073/pnas.1311542110. 24043791 PMC3791741

[64] Yount B, Roberts RS, Lindesmith L, Baric RS 2006 Rewiring the severe acute respiratory syndrome coronavirus (SARS-CoV) transcription circuit: engineering a recombination-resistant genome. Proc Natl Acad Sci U S A 103:12546–12551. doi:10.1073/pnas.0605438103. 16891412 PMC1531645

[65] Sims AC, Tilton SC, Menachery VD, Gralinski LE, Schäfer A, Matzke MM, Webb-Robertson BJ, Chang J, Luna ML, Long CE, Shukla AK, Bankhead AR, Burkett SE, Zornetzer G, Tseng CT, Metz TO, Pickles R, McWeeney S, Smith RD, Katze MG, Waters KM, Baric RS 2013 Release of severe acute respiratory syndrome coronavirus nuclear import block enhances host transcription in human lung cells. J Virol 87:3885–3902. doi:10.1128/JVI.02520-12. 23365422 PMC3624188

[66] Sheahan T, Rockx B, Donaldson E, Corti D, Baric R 2008 Pathways of cross-species transmission of synthetically reconstructed zoonotic severe acute respiratory syndrome coronavirus. J Virol 82:8721–8732. doi:10.1128/JVI.00818-08. 18579604 PMC2519660

